# Training Load and Post-Exercise Recovery Following Varied Game Intervals in 3x3 Basketball

**DOI:** 10.5114/jhk/203324

**Published:** 2025-04-30

**Authors:** Rūtenis Paulauskas, Paulius Kamarauskas, Bruno Gonçalves, Bruno Figueira

**Affiliations:** 1 Educational Research Institute, Education Academy, Vytautas Magnus University, Kaunas, Lithuania.; 2 Department of Sport and Health, School of Health and Human Development, University of Évora, Évora, Portugal.; 3 Comprehensive Health Research Centre (CHRC), University of Évora, Évora, Portugal.

**Keywords:** performance analysis, physical demands, tracking technology, rate

## Abstract

The study aimed (I) to quantify external and internal training loads during simulated 3x3 basketball games of 1, 2, 3, and 4 min, each followed by 1-min rest intervals; and (II) to assess the heart rate and muscle oxygen saturation recovery rates following, 1-, 2-, 3-, and 4-min periods of play. Twelve (n = 12) elite male 3x3 basketball players (age: 26.6 ± 5.6 years, body height: 196.8 ± 5.5 cm, body mass: 92.0 ± 9.5 kg, training experience: 15.4 ± 3.9 years, and 3x3 basketball training experience: 4.8 ± 1.6 years) from the Lithuanian national team participated in two controlled simulated games. Differences between playing intervals were analysed using a repeated-measures factorial analysis of variance. The results demonstrated that extended 3x3 basketball play duration led to significant increases in total distance covered, the sprint count, and the jump count, while diminishing dynamic responses in the deceleration rate, average speed, the jump rate, and high-intensity effort. Notably, SmO_2_ levels exhibited stability during recovery intervals regardless of activity duration, whereas heart rate recovery improved markedly, particularly following extended play periods. This knowledge is instrumental in refining training protocols for 3x3 basketball, emphasizing the necessity of recovery strategies tailored to specific play duration.

## Introduction

Three-on-three (3x3) basketball is distinguished by its fast-paced nature and intense competition schedules, ranking among the most demanding sports globally. The sport’s unique rules are designed to maximize its spectacle, requiring exceptional effort, preparedness, and skill from both players and coaching staff. Despite its inclusion in the Olympic Games, there has been a notable lack of research over the past decade regarding the profiles of 3x3 basketball players and the specific demands of the game (Cabarkapa et al., 2023, 2024; Montgomery and Maloney, 2018; Schmitz et al., 2024).

Many 3x3 players either concurrently engage in 5x5 basketball or have developed their foundational skills within this traditional format. Significant differences exist between these formats in terms of tactical, physical, and physiological performance metrics (Cabarkapa et al., 2024; Erculj et al., 2019; Figueira et al., 2022). Additional research is essential to provide a deeper, evidence-based understanding of the demands specific to 3x3 basketball. The emergence of GPS (Global Positioning System) and LPS (Local Positioning System) tracking technologies has provided researchers and practitioners with valuable tools to accurately assess the internal and external loads experienced by athletes in 3x3 basketball contexts. During the competitive phase, male players exhibited increased engagement in high-intensity activities, specific high-intensity movements, and jumping, but allocated more playing time to jumping and recovery activities (e.g., standing or walking) during final matchups compared to group stage games (Ferioli et al., 2023). These findings underscore the significance of anaerobic endurance as a critical energy source in 3x3 basketball, while emphasizing the importance of aerobic capacity for facilitating rapid player recovery during brief recovery periods.

To qualify for the Olympics and World Tour Finals and accumulate ranking points, players must contend with a rigorous six-month 3x3 competition period. This demanding schedule requires participation in weekly tournaments, enduring extensive travel and managing the effects of jet lag, all of which necessitate meticulous preparation to effectively handle stress and recovery. The advent of the comprehensive FIBA physical preparation guide has revolutionized the approach to periodization in 3x3 basketball, emphasizing the optimization of muscle energy resources, player load management, and strategic exercise selection (*Physical-Preparation-of-Professional-3x3-Athletes.Pdf*, n.d.). In 3x3 basketball games, where players are responsible for making tactical decisions autonomously, effective player rotation and timely substitutions are crucial for sustaining high-intensity performance intervals. Analysis of the World Tour Final reveals that players typically engage in play for a time ranging from 30 to 75 s (54% of observed intervals), while they spend 15 to 30 s on the bench (45% of observed intervals).

Additionally, there are notable instances where players are actively engaged for 76 to 180 s (28% of observed intervals), and spend 31 to 60 s on the bench (24% of observed intervals) (*Physical-Preparation-of-Professional-3x3-Athletes.Pdf*, n.d.). Various factors, including performance development, tactical strategies, and player injuries, can significantly influence the duration and dynamics of player rotation in a basketball game (Sosa-Martin et al., 2024). This underscores the relevance of several critical research questions related to in-game recovery (Vermeulen et al., 2024). Bishop et al. (2008) classify recovery between work intervals as short-term recovery, which primarily involves the resynthesis of phosphocreatine (PCr). From both physiological and gameplay perspectives, the heart rate (HR) is commonly employed as an indicator to quantify training loads and recovery demands (Heidari et al., 2019).

Near-infrared spectroscopy (NIRS) which measures muscle oxygen saturation (SmO_2_), has emerged as a cost-effective and accessible technology for sports performance analysis. NIRS provides valuable real-time monitoring of skeletal muscle oxygenation, complementing data on external load and the HR. This allows coaches and conditioning trainers to make informed decisions regarding training and recovery protocols (Feldmann et al., 2019; Perrey and Ferrari, 2018). NIRS facilitates the evaluation of interventions aimed at optimizing the balance between oxygen delivery and utilization, a critical factor influencing muscle performance (Paulauskas et al., 2022). Available research has demonstrated that different forms of repeated sprint exercises elicit distinct physiological responses and metabolic demands in highly trained basketball athletes (Figueira et al., 2021), with SmO_2_ levels dropping to 40% during sprints and recovering to approximately 80% during passive recovery phases (Paulauskas et al., 2020). Consequently, the intensity experienced during a 3x3 game, and the duration of rest intervals can lead to varied physiological responses, ultimately impacting the game outcome. Analysing these specific components would enable coaches to refine training regimens, effectively design training schedules, and promote the development of 3x3 basketball players.

The provided information facilitated the simulation of a 3x3 basketball game, in which the most frequent intervals of activity were interspersed with the most common periods of rest. To date, our understanding of the strain induced by varying duration of 3x3 game intervals remains incomplete. Moreover, the current game intervals lack quantification, thereby hindering the development of performance benchmarks for elite players. The varying strain provokes a distinct acute response and modifies recovery dynamics, both of which are critical for competitive success.

Thus, the objectives of our case study were (I) to quantify external and internal training loads during simulated 3x3 basketball games of 1, 2, 3, and 4 min, each followed by 1-min rest intervals; and (II) to assess the HR and SmO_2_ recovery rates following, the 1^st^, the 2^nd^, the 3^rd^, and the 4^th^ min of play. Based on the dynamics of 3x3 gameplay, we hypothesized that 1 min of recovery would yield varying levels of physiological recovery depending on the preceding duration of activity. Additionally, we hypothesized that the recovery status would manifest in diminished relative movement performance characteristics.

## Methods

### Participants

Twelve (n = 12) professional male 3x3 basketball players (age: 26.6 ± 5.6 years, body height: 196.8 ± 5.5 cm, body mass: 92.0 ± 9.5 kg, training experience: 15.4 ± 3.9 years, and 3x3 basketball training experience: 4.8 ± 1.6 years) from the Lithuanian national team were recruited for this study. The selected participants were 3^rd^ place winners in the 3x3 Europe CUP 2022, 3^rd^ place winners in the 3x3 basketball competition at the 2024 Summer Olympic Games, and 3^rd^ place winners in the 3x3 Europe CUP 2024. All athletes were cleared by medical and coaching staff to participate in team training activities and competition. The case study received ethical approval from the Institutional Ethics Committee of the Vytautas Magnus University (approval code: SA-EK-21-04; approval date: 30 November 2021) in accordance with the Declaration of Helsinki guidelines.

### Design and Procedures

An observational study was conducted during the preparation phase for the 2024 Summer Olympic Games and the 2024 European CUP. A total of two simulated 3x3 basketball games were played during the data collection period, with each player participating in one simulated game. The simulated 3x3 basketball games were played outside on standard FIBA approved 3x3 flooring designed for outdoor play (15 m width, 11 m length, 3.05 m basket height), under the sunny weather conditions (air temperature 23.5 ± 3.5^°^C, wind speed 3.1 ± 0.8 m/s). Prior to each game, players completed a 20-min warm-up session, which included dynamic stretching and specific basketball-based movement exercises, under the supervision of a strength and conditioning coach. The simulated games were structured into four playing periods of varying duration (1, 2, 3, and 4 min), with 1 min of passive recovery. The official 3x3 basketball rules (https://fiba3x3.com/docs/fiba-3x3-basketball-rules-full-version.pdf) were modified by removing free-throw shots and substitutions, and by incorporating 1-min recovery periods between each playing period. These adjustments were made to replicate the actual playing conditions (substitution of players and resting on the bench) and to assess HR and SmO_2_ recovery rates, following different durations of 3x3 basketball play.

### Data Collection and Processing

External load variables were measured using VXSport inertial measurement devices (VXSport, Wellington, New Zealand), which sampled data at a rate of 100 Hz (Smith et al., 2024). The VXSport units were placed between the scapulae using the manufacturer’s purpose-designed vest, ensuring a secure fit prior to the simulated 3x3 basketball games. These triaxial devices, equipped with gyroscopes, accelerometers, and magnetometers capture movement changes in all directions. VXSport devices were used to measure both the volume and intensity of the external load. Total distance (TD) (m), sprints (ST) (n), and the jump count (n) were selected as variables of external load volume. For external load intensity, the following variables were assessed: a distance rate (DR m/min), average speed (speed_avg_, km/h), maximum speed (speed_max_, km/h), a jump rate (JR, jumps/min), and average height jump zone total (AHJZT, cm) (Smith et al., 2024).

VXSport devices were also employed to analyse continuous HR measurements, using Suunto HR sensors (Suunto Smart sensor, Suunto, Oy, Finland) (Smith et al., 2024). These sensors were integrated into a manufacturer-designed vest compatible with strapless HR monitoring. The HR devices were positioned between the scapulae, next to the VXSport inertial measurement devices. This purpose-designed vest, with integrated HR sensors, allowed for strapless HR measurements. The HR data were stored on the VXSport devices via Bluetooth connection. The collected HR data were used to calculate the average HR (HR_avg_, bpm), and the maximum HR (HR_max_, bpm) during play periods and the resting HR (rest HR, bpm) during recovery periods.

Following data collection, the external load and HR data were downloaded, stored, and processed using VXSport software (VXSport, VXSport release 7.1.0.4). The processed data were then exported for further analysis and calculations.

Near-infrared spectroscopy (NIRS) measurements were conducted using the Moxy monitor devices (Moxy, Fortiori Design LLC, Minnesota, USA) (Paulauskas et al., 2020). The Moxy device operates by sequentially emitting light waves (630–850 nm) from four light-emitting diodes into the underlying tissue and recording the amount of scattered light returning at two detectors positioned 12.5 and 25 mm from the light source.

The depth of light penetration corresponded to half the distance between the light source and the detector. The scattered light was processed by an algorithm that integrated a tissue light propagation model and the Beer-Lambert law to quantify the absorption of light at wavelengths associated with oxygenated and deoxygenated hemoglobin (Hb).

Moxy devices were positioned on the participant’s dominant leg, specifically on the vastus lateralis muscle, halfway between the greater trochanter and the lateral epicondyle of the femur. Before placement, the area was trimmed with an electric razor (if necessary) and cleaned with alcohol swabs. The device was secured with a light shield and athletic tape to prevent ambient near-infrared light from interfering with the detectors. SmO_2_ was continuously monitored throughout the simulated 3x3 basketball game, and the data were used to calculate average SmO_2_ (avg SmO_2_, %) during playing periods and resting SmO_2_ (rest SmO_2_, %) during recovery periods. After data collection, SmO_2_ data were downloaded for further calculations.

To assess the metabolic-anaerobic demand in athletes, blood lactate (BLa) concentration (mmol/L) was measured 3 min post-completion of simulated 3x3 basketball game (Paulauskas et al., 2020). Blood lactate samples were collected from the fingertips of each participant and immediately analysed using a validate lactate analyser (Lactate Pro, Arkray, Tokyo, Japan).

The HR and SmO_2_ relative (%) delta changes (Δ) during recovery phases were utilized as key metrics for evaluating recovery indices. These changes were quantified by subtracting values recorded at the 15^th^, the 30^th^, the 45^th^, and the 60^th^ s as a percentage of the game stop signal, which was defined as the HR and SmO_2_ measurements at the initiation of the recovery period (Dupuy et al., 2012). Recovery indices were calculated for each 1-min recovery period, following playing intervals of 1, 2, 3, and 4 min during a 3x3 basketball game.

### Statistical Analysis

A descriptive analysis was conducted, providing means and standard deviations for the dataset. The normality of the data distribution was assessed using the Shapiro-Wilk test. Group comparison was performed using a mixed linear model for repeated measures, incorporating both fixed and random effects. The random factor accounted for inter-subject variability, while intraclass correlation coefficients were calculated to evaluate the reliability of repeated measures. Marginal and conditional *R*2 values were provided to explain variance attributable to fixed effects and the entire model, respectively. To estimate the strength of significant findings, effect sizes (ES) were determined using the Cohen’s *d*. Effect size values were interpreted as follows: < 0.20 represented a trivial effect, 0.20 to 0.49 was classified as a small effect, 0.50 to 0.79 corresponded to an intermediate effect, and 0.80 and higher was considered a large effect (Cohen, 1988; Cumming, 2017). All data sets were tested for each statistical technique corresponding assumptions. Statistical analyses were performed using SPSS (Version 20 for Mac; SPSS Inc., Chicago, IL, USA) and statistical significance was set at *p* < 0.05.

## Results

**Figure 1:**
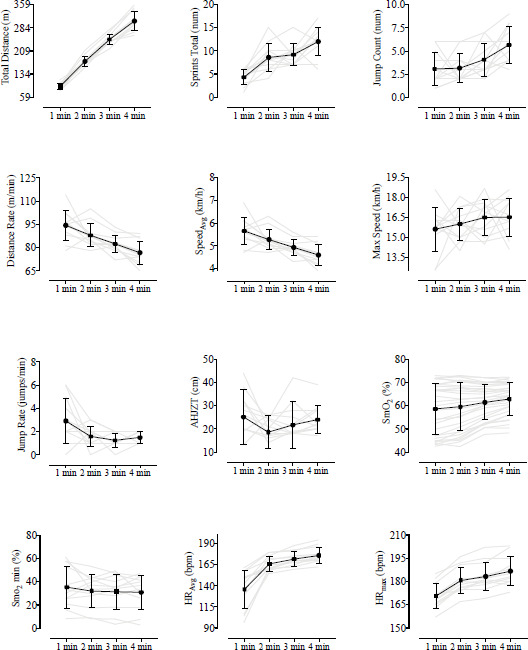
Descriptive analysis of performance variables during different bouts. The grey lines indicate the individual values for each athlete, while the black line denotes the mean value for each variable. Abbreviations: m = meters; num = number; min = minute; AHJZT = Average Height Jump Zone Total; cm = centimetres; SmO_2_ Rest = Muscle oxygen saturation; min = minimum; HR_Avg_ = Average Heart Rate; bpm = beats per minute; max = maximum

The results of the study provide a comprehensive analysis of various performance variables and recovery dynamics in 3x3 basketball players following 1, 2, 3, and 4 min of activity (Figure 1 and Table 1).

Players exhibited a significant increase in TD, across all intervals (*p* < 0.001), indicating a consistent increase in distance with longer intervals. Significant differences were found primarily in 1-min versus other intervals, suggesting a rapid increase in the sprint count in shorter intervals. DR, average speed, and high-intensity effort rate decreased as the duration of play increased. These decreases were statistically significant (*p* < 0.001). Considering the heart rate, both average and maximum heart rates (HR_avg_ and HR_max_) exhibited significant increases across intervals (*p* < 0.001), with notable distinctions between the 1-min and subsequent intervals.

**Table 1:** Training load according different game intervals.

Variable	1 min	%CV	2 min	%CV	3 min	%CV	4 min	%CV
TD (m)	95.2 ± 10.1	10.6	175.4 ± 15.1	8.6	246.7 ± 16.4	6.6	306.3 ± 30.5	10.0
ST (num)	4.33 ± 1.6	37.3	8.6 ± 2.9	34.2	9.2 ± 2.3	25.4	12.0 ± 3.1	25.4
JC (num)	2.9 ± 1.9	67.7	3.0 ± 18	58.6	3.8 ± 2.1	55.4	5.7 ± 2.0	34.8
DR (m/min)	94.3 ± 9.6	10.2	87.9 ± 7.4	8.4	82.3 ± 5.5	6.7	76.7 ± 7.5	9.8
Speed_Avg_ (km/h)	5.7 ± 0.6	10.4	5.3 ± 0.4	8.5	4.9 ± 0.3	6.8	4.6 ± 0.5	9.9
Speed_max_ (km/h)	15.6 ± 1.7	10.7	16.0 ± 1.2	7.5	16.5 ± 1.4	8.2	16.5 ± 1.4	8.7
JR (jumps/min)	2.9 ± 2.0	67.7	1.6 ± 0.9	56.9	1.3 ± 0.6	49.7	1.5 ± 0.5	34.8
AHJZT (cm)	25.2 ± 11.7	46.7	18.6 ± 7.1	38.2	21.8 ± 10.0	46.0	24.0 ± 60	25.1
HR_avg_ (bpm)	136.0 ± 22.6	16.6	165.8 ± 8.7	5.3	171.5 ± 8.5	5.0	175.8 ± 9.3	5.3
HR_max_ (bpm)	170.6 ± 8.1	4.7	180.6 ± 8.5	4.7	183.2 ± 9.1	5.0	186.6 ± 9.4	5.0
SmO_2avg_ (%)	53.2 ± 2.4	23.2	49.3 ± 12.1	24.6	51.4 ± 10.4	20.2	49.3 ± 6.7	13.5
SmO_2min_ (%)	35.3 ± 18.0	50.8	31.9 ± 14.3	44.7	31.4 ± 14.9	47.4	31.0 ± 14.7	47.6
Bla (mmol•L^−1^)	-	-	-	-	-	-	11.7 ± 5.3	45.2

Abbreviations: TD = Total Distance; m = meters; ST = Sprints Total; num = number; JC = Jump Count; DR = Distance Rate; min = minute; Avg = Average; km = kilometre; h = hour; JR = Jump Rate; AHJZT = Average Height Jump Zone Total; cm = centimetres; HR = Heart Rate; bpm = beats per minute; max = maximum; SmO_2_ = Muscle oxygen saturation; Bla = blood lactate concentration; mmol•L^−1^ = millimoles per litre; %CV = Coefficient of Variation

**Table 2:** Model Summary.

Variable	R-Squared		Random Components				Random effect LRT		
	Marginal	Conditional	Variance	Variance 95% CI		ICC	AIC	LRT	*p*
				Lower	Upper				
TD (m)	0.94	0.97	173	49.7	473	0.454	406	10.5	0.001
ST (num)	0.54	0.61	0.91	0.00	4.06	0.140	227	1.12	0.290
JC (num)	0.25	0.45	1.03	0.03	3.36	0.267	204	3.78	0.052
DR (m/min)	0.43	0.64	22	4.68	63.4	0.376	324	7.26	0.007
Speed_avg_ (km/h)	0.43	0.64	0.08	0.02	0.231	0.368	77.4	6.97	0.008
Speed_max_ (km/h)	0.07	0.13	0.15	0.00	1.02	0.07	176	0.33	0.568
JR (jumps/min)	0.24	0.37	0.22	0.00	0.91	0.165	158	1.52	0.218
AHJZT (cm)	0.07	0.41	29.7	5.96	86.6	0.366	338	6.88	0.009
HR_avg_ (bpm)	0.57	0.78	91.1	28.3	244	0.488	375	12.2	<0.001
HR_max_ (bpm)	0.32	0.95	71.13	31.5	166.05	0.925	336	70.7	<0.001
SmO_2avg_ (%)	0.78	0.92	120.4	71.3	188.43	0.579	424	22.4	<0.001
SmO_2 min_ (%)	0.01	0.82	196.7	83.6	468.9	0.816	386	42.3	<0.001

Average SmO_2_ decreased slightly across the intervals, while the average HR increased. These trends were significant (*p* < 0.001).

Regarding recovery dynamics, SmO_2_ showed minimal variation between different recovery intervals. Changes were not statistically significant (*p* > 0.05) across the measured periods, indicating that SmO_2_ recovery remained relatively stable regardless of the duration of the preceding activity. HR recovery exhibited significant differences between intervals, particularly after 30 to 60 s of recovery (*p* < 0.001). HR recovery was more pronounced after 4 min of play, with a notable reduction compared to shorter play duration (Tables 3 and 4).

**Table 3:** Results of the mixed linear model for repeated measures for each bout and post-hoc analysis and mean changes with ±95% confidence limits.

Variable	F	*p*	1 vs. 2 min	1 vs. 3 min	1 vs. 4 min	2 vs. 3 min	2 vs. 4 min	3 vs. 4 min
			Post-Hoc Bonferroni					
TD (m)	478	<0.001	t = −13.6 *p* ≤ 0.001	t = −25.7 *p* ≤ 0.001	t = −35.8 *p* ≤ 0.001	t = −12.1 *p* ≤ 0.001	t = −22.2 *p* ≤ 0.001	t = −10.1 *p* ≤ 0.001
ST (num)	21.6	<0.001	t = −4.5 *p* ≤ 0.001	t = −5.0 *p* ≤ 0.001	t = −8.0 *p* ≤ 0.001	t = −0.6 *p* = 1.000	t = −3.5 *p* = 0.007	t = −2.9 *p* = 0.036
JC (num)	6.94	<0.001	t = −0.1 *p* = 1.000	t = −1.3 *p* = 1.000	t = −4.0 *p* = 0.002	t = −1.2 *p* = 1.000	t = −3.9 *p* = 0.003	t = −2.7 *p* = 0.069
DR (m/min)	18.9	<0.001	t = 2.6 *p* = 0.082	t = 4.9 *p* ≤ 0.001	t = 7.2 *p* ≤ 0.001	t = 2.3 *p* = 0.181	t = 4.6 *p* ≤ 0.001	t = 2.3 *p* = 0.168
Speed_avg_ (km/h)	18.2	<0.001	t = 2.5 *p* = 0.109	t = 4.8 *p* ≤ 0.001	t = 7.0 *p* ≤ 0.001	t = 2.3 *p* = 0.160	t = 4.5 *p* ≤ 0.001	t = 2.2 *p* = 0.205
Speed_max_ (km/h)	1.20	0.326	t = −0.7 *p* = 1.000	t = −1.6 *p* = 0.753	t = −1.6 *p* = 0.693	t = −0.9 *p* = 1.000	t = −0.9 *p* = 1.000	t = −0.0 *p* = 1.000
JR (jumps/min)	6.01	0.002	t = 3.1 *p* = 0.025	t = 3.9 *p* = 0.003	t = 3.3 *p* = 0.015	t = 0.8 *p* = 1.000	t = 0.2 *p* = 1.000	t = −0.6 *p* = 1.000
AHJZT (cm)	1.96	0.140	t = 2.2 *p* = 0.189	t = 1.2 *p* = 1.000	t = 0.4 *p* = 1.000	t = −1.1 *p* = 1.000	t = −1.8 *p* = 0.441	t = −0.8 *p* = 1.000
HR_avg_ (bpm)	40.6	<0.001	t = −7.5 *p* ≤ 0.001	t = −8.9 *p* ≤ 0.001	t = −10.0 *p* ≤ 0.001	t = −1.4 *p* = 0.992	t = −2.5 *p* = 0.104	t = −1.1 *p* = 1.000
HR_max_ (bpm)	98.2	<0.001	t = −10.2 *p* ≤ 0.001	t = −12.8 *p* ≤ 0.001	t = −16.3 *p* ≤ 0.001	t = −2.6 *p* = 0.077	t = −6.1 *p* ≤ 0.001	t = −3.5 *p* = 0.009
SmO_2 avg_ (%)	98.8	<0.001	t = −10.2 *p* ≤ 0.001	t = −12.8 *p* ≤ 0.001	t = −16.3 *p* ≤ 0.001	t = −8.6 *p* = 0.04	t = −6.1 *p* ≤ 0.001	t = −7.5 *p* = 0.009
SmO_2 min_ (%)	1.07	0.374	t = 1.2 *p* = 1.000	t = 1.5 *p* = 0.917	t = 1.6 *p* = 0.720	t = 0.2 *p* = 1.000	t = 0.3 *p* = 1.000	t = 0.1 *p* = 1.000

Abbreviations: TD = Total Distance; m = meters; ST = Sprints Total; num = number; JC = Jump Count; DR = Distance Rate; min = minute; Avg = Average; km = kilometre; h = hour; JR = Jump Rate; AHJZT = Average Height Jump Zone Total; cm = centimetres; HR = Heart Rate; bpm = beats per minute; max = maximum; max = maximum; SmO_2_ = Muscle oxygen saturation; Bla = blood lactate concentration; mmol•L^−1^ = millimoles per litre

**Table 4:** Relative Δ % changes in recovery rate during 1-min recovery.

Recovery rate	Variables	After 1 min	After 2 min	After 3 min	After 4 min
Δ 15%	SmO_2_	2.17 ± 7.78	5.08 ± 12.47	6.75 ± 8.47	7.75 ± 10.30
	HR	−3.30 ± 2.47	−0.61 ± 1.43	−0.28 ± 2.90	1.35 ± 1.21
Δ 30%	SmO_2_	16.08 ± 18.43	19.08 ± 7.35	14.42 ± 15.05	16.08 ± 11.07
	HR	−11.35 ± 4.89	−5.08 ± 2.69	−2.57 ± 3.04	0.86 ± 2.94
Δ 45%	SmO_2_	24.58 ± 17.18	25.57 ± 13.83	14.42 ± 15.05	19.11 ± 10.31
	HR 45	−21.58 ± 8.68	−15.13 ± 5.26	−10.33 ± 5.58	−4.01 ± 1.81
Δ 60%	SmO_2_	27.83 ± 18.08	30.17 ± 18.00	14.42 ± 15.05	23.94 ± 10.60
	HR	−27.43 ± 9.12	−21.20 ± 5.94	−15.52 ± 8.37	−8.61 ± 3.99

Note. Data of HR and SmO_2_ at 0 s are presented as absolute values. Variable changes at the 15^th^, the 30^th^, the 45^th^ and the 60^th^ s are presented as delta change from the game stop signal to 15, 30, 45 and 60 s. Abbreviations: SmO_2_ = Muscle oxygen saturation; HR = Heart Rate

**Table 5:** Model Summary.

Recovery rate	Variable	R-Squared		Random Components				Random effect LRT		
		Marginal	Conditional	Variance	Variance 95% CI		ICC	Marginal	Conditional	Variance
					Lower	Upper				
Δ 15%	SmO_2_	0.04	0.04	0.0	Δ 15%	SmO_2_	0.04	0.04	0.0	Δ 15%
	HR	0.39	0.39	0.0	0.00	HR	0.39	0.39	0.0	1.000
Δ 30%	SmO_2_	0.02	0.20	34.0	Δ 30%	SmO_2_	0.02	0.20	34.0	Δ 30%
	HR	0.63	0.63	0.0	0.00	HR	0.63	0.63	0.0	1.000
Δ 45%	SmO_2_	0.09	0.42	74.9	Δ 45%	SmO_2_	0.09	0.42	74.9	Δ 45%
	HR	0.55	0.63	6.4	0.00	HR	0.55	0.63	6.4	0.175
Δ 60%	SmO_2_	0.13	0.57	126.0	Δ 60%	SmO_2_	0.13	0.57	126.0	Δ 60%
	HR	0.49	0.75	25.9	8.33	HR	0.49	0.75	25.9	<0.001

Abbreviations: F = F ratio; p = between group-subject effect; η_p_^2^ = effect size

**Table 6:** Results of the mixed linear model for repeated measures for each bout of recovery rate and post-hoc analysis and mean changes with ±95% confidence limits.

Recovery rate	Variable	F	*p*	1 vs. 2 min	1 vs. 3 min	1 vs. 4 min	2 vs. 3 min	2 vs. 4 min	3 vs. 4 min
				Post-Hoc Bonferroni					
Δ 15%	SmO_2_	0.73	0.541	t = 0.7 *p* = 1.000	t = 1.1 *p* = 1.000	t = 1.4 *p* = 1.000	t = 0.4 *p* = 1.000	t = 0.7 *p* = 1.000	t = 0.3 *p* = 1.000
	HR	9.87	< 0.001	t = 3.1 *p* = 0.023	t = 3.5 *p* = 0.009	t = 5.4 *p* ≤ 0.001	t = 0.4 *p* = 1.000	t = 2.3 *p* = 0.187	t = 1.9 *p* = 0.418
Δ 30%	SmO_2_	0.30	0.826	t = 0.6 *p* = 1.000	t = −0.3 *p* = 1.000	t = 0.0 *p* = 1.000	t = −0.9 *p* = 1.000	t = −0.6 *p* = 1.000	t = 0.3 *p* = 1.000
	HR	26.1	< 0.001	t = 4.4 *p* ≤ 0.001	t = 6.2 *p* ≤ 0.001	t = 8.6 *p* ≤ 0.001	t = 1.8 *p* = 0.535	t = 4.2 *p* ≤ 0.001	t = 2.4 *p* = 0.131
Δ 45%	SmO_2_	2.51	0.076	t = 0.2 *p* = 1.000	t = −2.2 *p* = 0.217	t = −1.2 *p* = 1.000	t = −2.4 *p* = 0.128	t = −1.4 *p* = 1.000	t = 1.0 *p* = 1.000
	HR	23.1	< 0.001	t = 3.0 *p* = 0.035	t = 5.1 *p* ≤ 0.001	t = 8.0 *p* ≤ 0.001	t = 2.2 *p* = 0.214	t = 5.1 *p* ≤ 0.001	t = 2.9 *p* = 0.041
Δ 60%	SmO_2_	4.74	0.007	t = 0.5 *p* = 1.000	t = −3.0 *p* = 0.033	t = −0.9 *p* = 1.000	t = −3.5 *p* = 0.008	t = −1.4 *p* = 1.000	t = 2.1 *p* = 0.253
	HR	30.7	< 0.001	t = 3.0 *p* = 0.028	t = 5.8 *p* ≤ 0.001	t = 9.2 *p* ≤ 0.001	t = 2.8 *p* = 0.054	t = 6.1 *p* ≤ 0.001	t = 3.4 *p* = 0.012

## Discussion

This case-study aimed to identify the external and internal training loads associated with simulation game intervals of 1, 2, 3, and 4 min, alongside 1-min rest periods. Additionally, it evaluated HR and SmO_2_ recovery dynamics. The key findings were that (I) extending the duration of game intervals in 3x3 basketball was associated with reduced physical intensity and a more variable acute physiological response; and (II) HR recovery exhibited significant variation across different intervals, whereas muscle reoxygenation rates remained consistent throughout all game intervals.

### Training Load Progression

This study represents the first attempt to quantify the physical performance variables during a simulated 3×3 basketball game across distinct intervals: 1 min, 2 min, 3 min, and 4 min. The findings reveal that DR, Speed_avg_, and the JR progressively decreased as play duration increased, while TD, ST, and JC volumes significantly increased. These changes had a corresponding impact on internal exercise variables such as SmO_2avg_, SmO_2min_, HR_avg_, as well as HR_max_ and Bla (Table 1), underscoring the intricate relationship between intensity and volume in 3x3 basketball.

Previous research demonstrated that members of the German national 3x3 team maintained an average DR of 64 m/min during an international tournament (Willberg et al., 2023). Our study, using distinct game intervals, exceeded this benchmark. Specifically, in the 4-min interval, DR was lower than in shorter intervals, with an average of 76.67± 7.52 m/min. This difference might reflect the higher skill level of our participants or unique game dynamics, including recovery strategies. DR was also correlated with both speed_avg_ (km/h) and speed_max_ (km/h), both of them decreased as the exercise duration extended. Speed is a critical performance metric in basketball, reflecting overall physical capacity (*Fitness-Requirements-of-3x3-Players.Pdf*, n.d.). However, in-game velocity is often constrained by tactical roles, which vary with factors such as the playing style, the position, and game outcomes (Buchheit and Laursen, 2013), alongside limitations imposed by court dimensions and space availability. These tactical limitations can attenuate the relationship between a player’s maximal speed capacity (measured in lab or field settings) and their actual game performance (Mendez-Villanueva et al., 2011). Our comparison of in-game speed with controlled test results demonstrated that Speed_max_ during the 4-min interval was 30% lower than previously established speed benchmarks (*Fitness-Requirements-of-3x3-Players.Pdf*, n.d.). This supports the argument that in-game speed is a more reliable indicator of physical performance in 3x3 basketball than test-derived maximal velocity estimates.

In terms of the JR, players averaged 2.92 ± 1.98 jumps/min during 1-min intervals, closely matching the rate reported at the 2019 FIBA 3x3 World Cup rate (3.16 ± 0.9 jumps/min) (Ferioli et al., 2023). In contrast, German team players averaged 1.8 ± 0.6 jumps/min during an international 3x3 tournament (Willberg et al., 2023). In our study, the JR declined to 1.59 ± 0.90 jumps/min after 2 min of play. The significant reduction in the JR during the 4-min interval, as compared to the 1-min interval, likely resulted from neuromuscular fatigue and the critical dependence on ATP-PCr resynthesis for explosive movements.

High-intensity exercise induces rapid PCr depletion-up to 60%–80% within the first 30 s of activity, with as much as 70% depleted within the first 12 s (Spencer et al., 2005). Prolonged high-intensity exertion can lead up to 89% depletion of PCr stores (Hirvonen et al., 1992), necessitating varying recovery time post-exercise. PCr resynthesis occurs in two phases: an initial rapid phase followed by a slower one, with recovery rates depending on the type of recovery (active or passive) (Spencer et al., 2005). The half-life for PCr resynthesis ranges from 21 to 57 s, depending on exercise intensity and volume (McMahon and Jenkins, 2002). Approximately 65% of PCr can be restored within 90 s of rest, while full recovery (less than 90%) may take up to 6 min (McMahon and Jenkins, 2002). Intense exercise also leads to a sharp increase in ATP hydrolysis, which can be impeded by reductions in pH and increases in ATP, PCr, citrate, and free fatty acids. Elevated levels of ATP and PCr indicate a recovery state, reducing the reliance on glycolytic pathways during the initial stages of game intervals (Hirvonen et al., 1992).

A decrease in AHJZT during the 2-min interval may suggest the onset of rapid muscle fatigue, whereas a significant increase over the 4-min interval could reflect glycogen replenishment through the anaerobic metabolism. This conclusion is corroborated by our test results which recorded a Bla concentration of 11.71 ± 5.29 mmol/L at the conclusion of the simulation game. Glycogen is as a primary energy source for exercise performed at intensities exceeding 50%–60% of VO_2max_ (maximal oxygen uptake) (Mul et al., 2015). Depletion of glycogen reserves can contribute to muscle fatigue during aerobic exercise or prolonged high- intensity anaerobic activity (Abernethy et al., 1990).

The HR is frequently used as a proxy to regulate exercise intensity in sports (Achten and Jeukendrup, 2003). Elite 3x3 athletes, as studied by Montgomery and Maloney (2018), exhibited a maximum HR of 198 bpm and an average HR of 164 bpm, with consistent values across games. In our research, a similar average HR was achieved after 2 min, with a maximum HR peaking at 187 ± 9.4 bpm. However, the HR may not fully capture physical exertion during very short (30 s) or moderate-length (1–2 min) exercise intervals (da Cunha et al., 2011), largely due to the delay in HR response relative to VO_2_ uptake (MacDougall et al., 1977). Muscle deoxygenation, which responds more rapidly than VO_2_, is driven by the oxygen demands of active muscles and modulates O_2_ delivery by the cardiovascular system (Hesford et al., 2013).

Metrics such as muscle deoxygenation and the HR are modulated by external variables such as TD, sprints, jumps, and speed. Both average and SmO_2_ min suggest a consistent peripheral impact on oxygen extraction across all exercise intervals. NIRS-based assessments provide a more precise evaluation of exercise demands (Perrey and Ferrari, 2018), highlighting individualized physiological responses to players' diverse on-field activities. Consequently, individual player assessments yield deeper insights compared to evaluating the cumulative workload of a team.

### Post-Exercise Recovery

**Figure 2:**
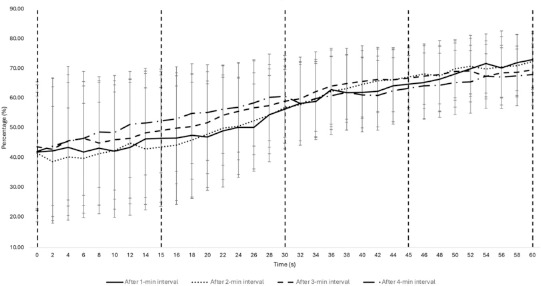
Fluctuations in SmO_2_ values during 60-s recovery periods, following 1, 2, 3, and 4 min of play in a 3x3 basketball game. Data are presented as absolute values (mean ± SD).

**Figure 3:**
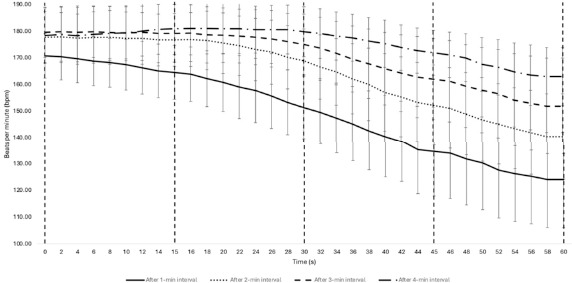
Fluctuations in HR values during 60-s recovery periods, following 1, 2, 3, and 4 min of play in a 3x3 basketball game. Data are presented as absolute values (mean ± SD).

This study explored post-exercise recovery dynamics by analysing HR and SmO_2_ indices during specific 3x3 game intervals, as illustrated in Figures 2 and 3. The HR-derived variables demonstrated notable variations across all four game intervals, indicating that recovery rates are influenced by exercise volume.

Following a 1-min play interval, the HR was observed to be at its lowest point immediately at the onset of recovery and after one minute of rest. In contrast, after the 2-, 3-, and 4-min play intervals, HR levels were elevated immediately post-exercise and exhibited varied recovery rates during the subsequent rest periods. The relative Δ HR values also fluctuated across different interval duration, underscoring the variability in recovery (Table 4). For instance, after a 1-min play interval, the recovery Δ 45% was −21.58 ± 8.68, closely mirroring the recovery Δ 60% after a 2-min interval, at −21.20 ± 5.94. Additionally, after a 1-min play interval, the recovery Δ 15% was −3.30 ± 2.47, comparable to the recovery Δ 45% post-4-min play at −4.01 ± 1.81. This pattern is likely attributable to the metabolic demands of anaerobic glycolysis and associated energy neuromuscular strain, contributing to varied fatigue levels (Buchheit and Laursen, 2013).

In athletes, HR recovery is primarily governed by the autonomic nervous system, specifically through parasympathetic reactivation and sympathetic withdrawal (Perini et al., 1989). The autonomic nervous system's interaction with other physiological systems, particularly reflected in HR recovery, is considered an indicator of the body’s capacity to manage exercise-induced stress (Facioli et al., 2021). Our investigation into HR recovery dynamics in basketball players, following different playing interval duration, supports this premise. Previous research has shown that Δ 60 was significantly higher after exercise at 70–80% VO_2max_ compared to 60% VO_2max_, implying a more rapid recovery in individuals with higher HR levels (Mann et al., 2014). However, the study by Mann et al. (2014) focused on submaximal exercises that involved relatively low anaerobic energy expenditure. Consequently, the lactate accumulation affecting HR recovery kinetics (Buchheit et al., 2007) may have been modest and less comparable to the physiological demands experienced during 3x3 basketball.

In 3x3 basketball, where only one reserve player is available, both work and rest duration is highly individualized. Recovery periods are often tailored by utilizing either a fixed value or a percentage of HR_max_ (Buchheit and Laursen, 2013). However, emerging evidence suggests that such an approach may not be particularly effective (Seiler and Hetlelid, 2005). During recovery, the HR is not directly linked to systemic O_2_ demand or muscle energy turnover (Buchheit et al., 2012), but instead correlates more strongly with the intensity of central command and metaboreflex activation (Rowell and O’Leary, 1990). Table 3 highlights the distinct dynamics between muscle reoxygenation and HR recovery, with the rates of relative recovery Δ SmO_2_ and Δ HR varying according to the work duration. In simulated play scenarios, specific training protocols facilitated quicker SmO_2_ recovery to pre-exercise levels.

Notably, reoxygenation levels across all work intervals remained consistent, as reflected in the uniform Δ SmO_2_ % recovery values across 1-, 2-, 3-, and 4-min intervals. Previous research has indicated that different exercise modalities do not significantly impact muscle reoxygenation (Paulauskas et al., 2020). Our findings suggest that O_2_ uptake recovery may be less dependent on exercise duration or the type of activity, and more related to the athlete’s ability to resist fatigue (SmO_2_% decrease) and replenish energy substrates (ATP, PCr), both of which are oxygen-dependent processes (Dupont et al., 2010).

There is strong concordance between recovery time constants derived from NIRS and PCr recovery time constants measured via magnetic resonance spectroscopy (Hamaoka et al., 1996), as well as a strong correlation with oxidative capacity assessed through muscle biopsy (Ryan et al., 2014). The similarity in muscle recovery levels may be influenced by factors such as neural drive efficiency, motor unit activation patterns, and metabolite accumulation (Glaister, 2005).

The results of this study suggest that varying training loads during 3x3 basketball do not significantly alter peripheral muscle oxygenation, and that SmO_2_ can serve as a reliable predictor of hemodynamic changes. Moreover, the decline in the HR is likely linked to parasympathetic reactivation, which reflects the body’s overall response to acute stress from exercise.

## Conclusions

The findings reveal that extending play duration (1, 2, 3, and 4 min) significantly increases external metrics such as TD, ST and JC, while reducing physical intensity markers such as DR, Speed_avg_, JR and AHJZT with no change in Speed_max_. This extension also specifically impacts internal physiological markers, increasing the HR_avg_ and the HR_max_ as well as variations in SmO_2avg_.

This study offers critical insights into the recovery dynamics of the HR and SmO_2_ in elite 3x3 basketball players across varying game intervals. Notably, HR recovery shows greater sensitivity to the length of play intervals compared to SmO_2_. While SmO_2_ levels remain relatively stable during recovery, HR recovery exhibits significant variability, particularly following the 4-min play interval. These results highlight the HR as a more responsive indicator of recovery status, reinforcing the need for tailored recovery protocols based on activity duration. These findings underscore the critical role of individualized recovery strategies in optimizing performance during high intensity 3x3 basketball games. Incorporating this knowledge into training regimens can enhance endurance, mitigate fatigue, and ultimately improve overall game performance. Furthermore, understanding these recovery dynamics can guide the optimal length and frequency of substitutions during games, particularly as players must make strategic decisions independently of direct coaching.

## Limitations

In assessing the metabolic demands of 1-, 2-, 3-, and 4-min play intervals, it is imperative to base findings on empirical data. A notable limitation, however, was the inability to measure Bla during the 1-min rest period, as the sampling process requires 3 min and would have compromised the integrity of the protocol. As a result, Bla measurements were only taken after the completion of the entire simulation game. Additionally, our study did not assess the potential impact of specific environmental conditions, which could further influence physical performance and recovery in outdoor sports settings. Future research should also investigate into the roles of psychological recovery and mental fatigue, as these elements are likely critical in shaping performance and recovery outcomes in high-intensity, rapid-paced sports such as 3x3 basketball.
